# A brief symptom index for advanced renal cell carcinoma

**DOI:** 10.1186/1477-7525-4-68

**Published:** 2006-09-26

**Authors:** David T Eton, David Cella, Jennifer Bacik, Robert J Motzer

**Affiliations:** 1 Center on Outcomes, Research and Education, Evanston Northwestern Healthcare, 1001 University Place, Suite 100, Evanston IL, 60201, USA; 2 Northwestern University Feinberg School of Medicine, Chicago, IL, USA; 3 Department of Epidemiology and Biostatistics, Memorial Sloan-Kettering Cancer Center, New York, NY, USA; 4 Genitourinary Oncology Service, Division of Solid Tumor Oncology, Memorial Sloan-Kettering Cancer Center, New York, NY, USA

## Abstract

**Background:**

Our objective was to test a brief, symptom index for advanced renal cell carcinoma, a disease affecting over 38,000 Americans each year and often diagnosed in late stages.

**Methods:**

We conducted secondary data analyses on patient-reported outcomes of 209 metastatic renal cell carcinoma patients participating in a Phase III clinical trial. Patient-reported outcomes, obtained from the FACT-Biological Response Modifier (FACT-BRM) scale, were available at baseline, 2, and 8 weeks. We analyzed data from eight FACT-BRM items previously identified by clinical experts to represent the most important symptoms of advanced renal cell carcinoma. Items comprising this index assess nausea, pain, appetite, perceived sickness, fatigue and weakness, with higher scores indicating fewer symptoms. We determined reliability and validity of the index and estimated a minimally important difference.

**Results:**

The index had excellent internal reliability at all three time points (alphas ≥ 0.83). Baseline scores were able to discriminate patients across Karnofsky performance status, number of metastatic sites, and risk group categories (ps < .01). Mean index scores declined over time likely indicative of the toxic nature of the administered treatments. Distribution- and anchor-based methods converged on a minimally important difference estimate of 2 to 3 points.

**Conclusion:**

The 8-item index of patient-reported symptoms of renal cell carcinoma appears to be a psychometrically sound measure. It is a brief, reliable, and valid measure that can easily be adapted for use in clinical trials and observational studies.

## Background

Renal cell carcinoma (RCC) is estimated to affect over 38,000 Americans annually and account for over 12,000 deaths [1]. It is often diagnosed in advanced stages, with varied sites of metastases. Due to uniform resistance to most cytotoxic chemotherapy agents [2], patients with metastatic RCC typically have very poor prognoses. Though not curative, biological response modifiers such as interleukin-2 and interferon-alfa have proven effective at delaying tumor growth and disease progression. However, these treatments can result in severe and sometimes dose-limiting toxicities such as fever, chills, nausea, vomiting, hypotension, and fatigue [3].

Recently, less-toxic targeted therapies have shown anti-tumor activity in clinical trials of metastatic RCC. Among the most promising agents are bevacizumab (Avastin), sorafenib (Nexavar) and sunitinib (Sutent). Bevacizumab, approved for the treatment of metastatic colorectal cancer in 2004, has also shown clinical activity in metastatic RCC [4]. Sorafenib, an oral multi-kinase inhibitor, has demonstrated anti-tumor activity in several solid tumors including RCC [5,6]. A recent Phase II trial demonstrating the efficacy of second-line sunitinib – a multi-targeted, tyrosine kinase inhibitor – has led to the rapid approval of this drug for the treatment of metastatic RCC [7]. Though better tolerated than immunotherapy, such targeted therapies have been associated with a host of toxicities including fatigue, diarrhea, nausea, dyspepsia, hypertension, proteinuria, rash, and malaise. Hence, regardless of treatment type, the clinical picture of advanced RCC features an array of symptoms and complications.

Measurement of symptoms and complications has often been done using common toxicity criteria, global measures of performance status, and/or formal quality of life assessment. Though the latter has proven highly effective at measuring subjective patient status in many functional areas, regulatory agencies and clinicians sometimes prefer briefer, symptom-focused instruments in clinical trials. To meet the need for brief and focused assessment of symptoms and complications associated with advanced RCC and its treatment, we used clinical experts to identify an 8-item index of questions from an available health-related quality of life (HRQL) questionnaire. Here we attempt to validate this index using data from a Phase III clinical trial. A secondary objective was to determine a minimally important score difference for the index.

## Methods

### Development of the index

The index of RCC symptoms and complications was developed in consultation with four medical oncologists with substantial expertise in the treatment of metastatic RCC. All were employed at large academic medical centers at the time of this study. These experts were shown a pool of items from a previously validated tool used to assess HRQL in patients treated with biological response modifiers – the Functional Assessment of Cancer Therapy – BRM (FACT-BRM) [8]. The FACT-BRM consists of 40 items, divided into 5 subscales: physical, functional, social, and emotional well-being and BRM-specific concerns. The first four subscales form the FACT-General, a measure of cancer-specific quality of life. The BRM concerns subscale consists of physical and mental concerns and issues relevant to patients receiving immunotherapies like interleukin-2 and interferon-alfa. Experts were asked to nominate a brief list of items from the FACT-BRM representing the clinical issues of greatest relevance in the advanced RCC setting. This could include symptoms associated with tumor burden as well as side effects of currently available or newly emerging treatment regimens. These experts agreed on the inclusion of the following eight items for the index:

- I have a lack of energy

- I have nausea

- I have pain

- I feel sick

- I am forced to spend time in bed

- I get tired easily

- I feel weak all over

- I have a good appetite

We maintained the 5-point ordinal rating scale of the original FACT-BRM, with responses ranging from 0 – 'not at all' to 4 – 'very much'. An aggregated symptom index score is created by first reverse-coding the negatively-worded items (all items except "I have a good appetite") then summing the responses to all 8 items so that a higher score indicates fewer symptoms and better well-being. Hence, the worst possible score is 0 and the best possible score is 32.

### Validation of the index

#### Data source

We conducted secondary data analyses on 209 advanced RCC patients with available HRQL data (70% male; mean age = 58.1 years) who were entered on a multi-center randomized Phase III trial comparing interferon with or without 13-cis-retinoic acid. Participating centers included Memorial Sloan-Kettering Cancer Center (54 patients) and member institutions of the Eastern Cooperative Oncology Group (155 patients). The trial was approved by the institutional review boards of each participating center. All patients provided informed consent. For more complete trial details see Motzer and colleagues (2000) [9]. Patients in the trial were required to have a pretreatment Karnofsky Performance Status greater than 60% and an estimated life expectancy of more than 3 months. Over half (53%) of patients in this analysis had a prior nephrectomy. The full 40-item FACT-BRM scale was used in the trial. For validation purposes, we extracted and scored the eight FACT-BRM items constituting the proposed symptom index. Assessments were conducted at baseline (prior to treatment) and at 2, 8, 17, 34, and 52 weeks after the initiation of therapy. Since completion rates fall well below 50% by 17 weeks, we considered only the baseline, 2 week, and 8 week data. Data were aggregated across treatment.

#### Data analyses

Internal reliability was determined by computing Cronbach's alpha coefficients and inter-item correlations at each of the three time points. Concurrent validity was determined by comparing baseline index scores across various clinical parameters using one-way analyses of variance (ANOVA) and independent sample t-test. Index scores were compared across Karnofsky performance status, number of identified metastatic sites, and prognostic risk group. These parameters were either directly available from clinical report forms or, in the case of risk group, determined from a composite of pretreatment features available on the forms. We determined responsiveness to change over time by comparing baseline to follow-up index scores using repeated-measures ANOVA. Least significant difference post-hoc tests were used to specify pairwise differences for any significant omnibus F-test. Finally, we computed an estimate for a minimally important difference (MID) for the index using both distribution- and anchor-based analyses [10]. The MID is the smallest difference in score likely to be clinically meaningful to patients and clinicians. Distribution-based measures included 1/3 and 1/2 of the standard deviation and one standard error of measurement (SEM). Anchor-based analyses compare index score differences between clinically distinct groups. Magnitude differences falling within an effect size range of 0.20 to 0.60 will be those considered minimally important. The effect size is computed as a mean difference between clinically distinct groups divided by the pooled within-group standard deviation.

## Results

### Reliability

Internal consistency reliability for the index was quite good in this sample of patients. Cronbach's alpha coefficients were 0.83, 0.86, and 0.86 at baseline, 2 weeks, and 8 weeks, respectively. Inter-item correlations demonstrate that the symptoms and complications represented in the items appear to co-vary with one another. After reverse-coding of negatively-worded items, the number of inter-item correlations reaching at least rho = 0.25 was 23 of 28 (82%) at baseline, 27 of 28 (96%) at 2 weeks, and 26 of 28 (93%) at 8 weeks. The number of inter-item correlations reaching at least rho = 0.50 was 7 of 28 (25%) at baseline, 10 of 28 (36%) at 2 weeks, and 11 of 28 (39%) at 8 weeks.

### Concurrent validity

Baseline index scores were compared across clinically distinct groups to determine concurrent validity. As shown in Table 1, these scores differentiated groups based on Karnofsky performance status (KPS), number of established metastatic sites and prognostic risk category. Patients with higher (better) performance status had higher index scores at baseline than did patients with lower (worse) performance status. In particular, patients with a KPS rating of 80 or 90 had higher mean index scores than did patients with a KPS rating of 70 (ps < .01). Though patients with a KPS rating of 90 had a higher mean baseline index score than patients with a KPS rating of 80, this difference was not statistically significant (p = .07). As shown in Table 1, patients with 0 or 1 metastatic site were compared to patients with multiple metastatic sites. Patients with 0 or 1 metastatic site prior to study treatment had significantly higher baseline index scores than patients with multiple metastatic sites prior to treatment (p < .01).

**Table 1:** Mean Index Scores By Baseline Clinical Parameters

	**Karnofsky rating**^2^				
	90 (n = 113)	80 (n = 45)	70 (n = 30)	F (post-hoc)^4^	Adjacent category mean differences & effect sizes
**Index score**^1^	24.76 (sd = 5.62)	22.89 (sd = 6.84)	18.34 (sd = 4.65)	F(2, 185) = 14.67*** 80,90 > 70	1.87 (0.30), 4.55 (0.73)
	**No. of metastatic sites**				

	0 or 1 site (n = 72)		2 or more sites (n = 116)	t	Mean difference & effect size
**Index score**	24.81 (sd = 5.57)		22.34 (sd = 6.42)	t(186) = 2.69**	2.47 (0.40)
	**Risk group**^3^				

	Favorable (F) (n = 47)	Intermediate (I) (n = 95)	Poor (P) (n = 41)	F (post-hoc)^4^	Adjacent category mean differences & effect sizes
**Index score**	26.96 (sd = 3.82)	23.35 (sd = 6.20)	19.47 (sd = 6.06)	F(2, 180) = 19.18*** F > I > P	3.61 (0.58), 3.88 (0.63)

Note. ^1^Higher score, indicates fewer symptoms and better well-being. ^2^90 = Able to carry on normal activity, minor signs or symptoms of disease. 80 = Normal activity with effort, some signs or symptoms of disease. 70 = Cares for self, unable to carry on normal activity or do active work. ^3^Based on pretreatment features associated with shorter survival. These include: low Karnofsky rating (< 80%), high lactate dehydrogenase level (≥ 1.5 times upper limit of normal), low hemoglobin level (≤ lower limit of normal), high corrected serum calcium (≥ 10 mg/dL), and absence of nephrectomy. Favorable risk = 0 risk factors present. Intermediate risk = 1–2 risk factors present. Poor risk = 3 or more risk factors present. ^4^Pairwise post-hoc comparisons made using least significant difference test.

**p < .01 ***p < .001

A previously developed prognostic factor model was used to define and categorize patients into risk groups. The model, derived from an analysis of 670 advanced RCC patients treated in clinical trials at the Memorial Sloan-Kettering Cancer Center between 1975 and 1996, classifies patients into risk groups based on pretreatment clinical features associated with shorter survival time [11]. These include: low performance status (KPS < 80), high lactate dehydrogenase (≥ 1.5 × 200 U/L – the upper limit of normal), low hemoglobin level (≤ the lower limit of normal: normal Hgb_male _= 13 g/dL and normal Hgb_female _= 11.5 g/dL), high corrected serum calcium (≥ 10 mg/dL), and absence of nephrectomy. Patients with 0 factors present are classified as favorable risk, those with 1 or 2 factors present are classified as intermediate risk, and those with 3 or more factors present are classified as poor risk. Baseline index scores were compared across these three risk groups (see Table 1). Patients with a favorable risk had significantly higher mean baseline index scores than did patients with either an intermediate or a poor risk (ps < .001). Patients with an intermediate risk had a higher mean baseline index score than patients with a poor risk (p < .001).

### Responsiveness to change

**Figure 1:**
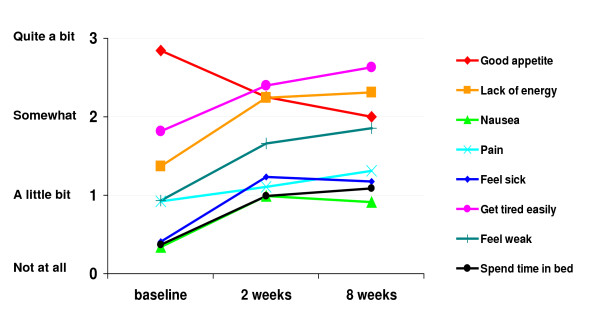
**Index item means at baseline, 2 weeks, and 8 weeks (complete cases)**^1^. ^1^Note. Number of complete cases fluctuates across item, ranging from n = 86 (feel sick) to n = 91 (lack of energy).

To determine whether items from the index are reflective of symptoms and complications that will change over the course of treatment for advanced RCC, we plotted mean scores of the eight individual items (see Figure 1). Consistent with the clinical profile of patients on interferon, many complications emerge upon the introduction of therapy. Mean levels of fatigue (lack of energy, getting tired, and feeling weak) increased from baseline to 2 weeks and did not fall at 8 weeks. Nausea, spending time in bed, and feeling sick – all low at baseline – rose moderately at 2 weeks and did not fall substantially at 8 weeks. Good appetite, high at baseline, declined some from baseline to 2 and 8 weeks. Pain, an important clinical indicator in metastatic disease, rose slightly over time. This pattern of fluctuation in the individual items justifies aggregation into a single index. A repeated-measures ANOVA was conducted on index scores across the three time points to determine responsiveness to change (complete cases only, n = 92). Index scores did change over time; F (2, 182) = 39.40, p < .001. Consistent with the changes observed in the individual items, the total index score dropped significantly from baseline (mean = 24.66, sd = 5.36) to 2 weeks (mean = 19.62, sd = 6.71) (p < .001) and remained significantly lower than baseline at 8 weeks (mean = 18.74, sd = 7.17) (p < .001).

### Minimally important difference

A minimally important difference (MID) was estimated by combining multiple distribution- and anchor-based analyses. The standard deviations of baseline, 2 week, and 8 week index scores were divided by 3 and 2 to determine 1/3 and 1/2 standard deviation (SD) estimates for an MID. Estimates based on 1/3 SD ranged from 2.07 to 2.38; estimates based on 1/2 SD ranged from 3.11 to 3.58. The SD-based criteria were supplemented by a second distribution-based parameter, the standard error of measurement (SEM). The SEM was computed at each of the three time points as, SD × (1 - reliability)^1/2^. SEMs for the three time points ranged from 2.56 to 2.68. Using these three distribution-based parameters, it appears that the MID for this index falls within the range of 2 to 3 points.

Anchor-based analyses were used to refine or confirm the MID estimate. Score differences between distinct groups were computed along with corresponding effect sizes. The baseline cross-sectional comparisons in the table were used to define these groups. Clinically distinct groups were defined as adjacent response categories for each of the clinical parameters used. For example, in analyses of Karnofsky performance status, patients with a KPS rating of 90 were compared to patients with a KPS rating of 80 and patients with a KPS rating of 80 were compared to patients with a KPS rating of 70. Comparing index scores of patients with a KPS rating of 90 to those with a KPS rating of 70 was *not *used since this contrast exceeds a minimal level of clinical distinction. These adjacent category differences in index scores as well as their corresponding effect sizes are shown in the last column of Table 1. Only the magnitude differences that fall within an effect size range of 0.20 to 0.60 were considered minimally important. This corresponds to a 'small' to 'medium' effect [12], which we have found to be a reasonable approximation of an MID on FACT measures [10]. Three of the five minimal group differences fell within this effect size range. These three mean differences ranged from 1.87 to 3.61. Combining these results with the results of the distribution-based analyses, we conclude that the MID on the index most likely falls within the range of 2 to 3 points.

## Discussion and conclusion

Our newly developed index for assessing symptoms and complications associated with advanced RCC performed well psychometrically. The index had excellent internal reliability across all three time points. The moderate to high inter-item correlations indicate that this set of items represents a constellation of coexisting symptoms. This is further borne out by the longitudinal changes in item scores. All symptoms tended to worsen from pretreatment to the on-therapy follow-up points. Hence, aggregating these eight items into a common index score appears justified and is in line with recent FDA guidance on the construction and use of patient-reported outcome measures (PROs) [13]. Supportive of its validity, baseline scores on the index differentiated clinical patient groupings based on Karnofsky performance ratings, number of metastatic sites, and prognostic risk. A combination of distribution and anchor-based analyses identified an MID estimate of 2 to 3 points.

There are a few noteworthy limitations to this study. First, the development of the index relied entirely on physician input. Results could have been different had input from patients also been sought. Future efforts to improve and refine the index would benefit from patient query. Second, some of the newer targeted, anti-cancer agents may result in toxicities (i.e., rash and diarrhea) that are not directly addressed in the current index. To address these issues, it may be possible to augment the index with a few items drawn from other established measures including those within the FACT measurement system (see review by Eton, Yost & Cella) [14]. However, any substantial modification to the existing index will require re-validation. Third, some of the group sample sizes in the baseline index comparisons are modest (N's < 50). This may limit the generalizability of the validation and MID results. Finally, we used only cross-sectional (baseline) data in the anchor-based analyses for determining the MID. Confirmation of the MID using longitudinal methods is warranted. Furthermore, replication of the MID analyses in other RCC samples, especially in patients receiving therapies different from those studied in this report, would enhance the generalizability of the estimate.

Based on these results this index would appear to be a reasonable choice as an endpoint in clinical studies of advanced RCC. It is a brief, clinically-derived measure with excellent psychometric properties. Many current and planned Phase II and III clinical trials in the RCC setting include therapeutic combinations in which an array of complications may emerge [6,15]. Some of these trials combine standard immunotherapy with newer targeted agents (e.g., bevacizumab, sorafenib, sunitinib). Others are directly comparing the clinical benefit of newer targeted therapies versus standard immunotherapy. Given this picture, symptom assessment will need to be broad enough to capture toxicities associated with *both *immuno- and targeted therapy as well as symptoms of metastatic disease. The index reported herein provides the clinical investigator with a good foundation for an assessment of PRO endpoints in metastatic RCC.

## Competing interests

The author(s) declare that they have no competing interests.

## Authors' contributions

DE participated in the design of the study, performed the statistical analysis and interpretation of data, and drafted the manuscript. DC participated in the design and conception of the study and helped revise the manuscript for important intellectual content. JB assisted with the acquisition and interpretation of data, and helped revise the manuscript for important intellectual content. RM assisted with data acquisition and helped revise the manuscript for important intellectual content. All authors have read and approved the final manuscript.
